# Supplementary material for: Demographic and genetic factors shape the epitope specificity of the human antibody repertoire against viruses
Source: Nat Immunol. 2026 Feb 16;27(3):600–12. doi: 10.1038/s41590-026-02432-7 (PMC12956577; doi:10.1038/s41590-026-02432-7)
Supplement: Supplementary file 1 — Supplementary note. [file 41590_2026_2432_MOESM1_ESM.pdf]

nature immunology

Supplementary information

https://doi.org/10.1038/s41590-026-02432-7

Demographic and genetic factors shape the epitope specificity of the human antibody repertoire against viruses

In the format provided by the authors and unedited

Supplementary Note

Effects of age, sex, and continent of birth are not confounded by batch effects

PhIP-seq data, like other techniques based on next-generation sequencing[1], can vary among sequencing batches and processing plates. To mitigate such technical effects, we corrected all VirScan *Z*-scores for plate effects, which also captured sequencing batch effects, using ComBat[2]. We first assessed the efficacy of our batch correction procedure, by performing principal component analysis of both uncorrected and corrected *Z*-scores from the MI (2,608 *Z*-scores) and EIP (3,210 *Z*-scores) datasets.

Prior to correction, MI samples formed two separated clusters on the first two principal components (PC1 and PC2) corresponding to different sequencing batches (Extended Data Fig. 2a), indicating substantial batch effects. However, no clustering was apparent after batch correction, and PC1 and PC2 were instead associated with age (Extended Data Fig. 2b,d), demonstrating that batch-corrected MI data is mainly affected by biological effects. As MI participants from the same age decade or with the same sex were not distributed randomly among batches, we then tested if age and sex effects are confounded by technical variation. To do this, we compared, for each peptide *Z*-score, the proportion of variance explained by technical batches before batch correction with the proportion of variance explained by age or sex, after batch correction. If biological effects are confounded by batch effects, we expect the two proportions to be correlated. However, we found weak correlations between the two statistics: antibody levels with the largest biological effects typically showed limited proportions of variance explained by batch effects, and *vice versa* (Pearson's coefficient $r_{\text{Age}} = 0.042$, Extended Data Fig. 2c; $r_{\text{Sex}} = -0.0063$, Extended Data Fig. 2e). Notably, peptides largely explained by batch effects were enriched for peptides attributed to the viral family 'Others', which includes several uncommon viruses, raising questions about the biological relevance of these peptides (Extended Data Fig. 2c,e). These results, together with the replication of age and sex associations with standard immunoassays or in previous studies (see Results section), provide strong evidence that age and sex effects on the antibody repertoire of MI participants are genuine.

We then conducted the same analyses in the EIP cohort, leveraged to compare the antibody repertoire of individuals born in Central Africa (AFB) and Europe (EUB). PCA of the uncorrected EIP data showed weaker clustering according to batches, relative to MI data (Extended Data Fig. 2f). After batch correction, EUB and AFB separated on PC1 and PC2 (Extended Data Fig. 2g), suggesting that variance in antibody reactivity in EIP participants is

driven by continent of birth effects. However, EUB and AFB were not randomly distributed among batches, with AFB being included in batches 1 and 3 only (Extended Data Fig. 2f,g). To verify that population differences are not confounded by technical variation, we again compared for all public peptide *Z*-scores the proportion of variance explained by batches before correction and that explained by continent of birth after correction. We observed a clear correlation between the two proportions, suggesting potential confounding ($r_{\text{Continent}}$ = 0.63, Extended Data Fig. 2h). However, for 89% of the *Z*-scores associated with continent of birth ($P_{\text{adj}} < 0.05$; Fig. 3a), the variance explained by continent of birth largely exceeded that explained by technical batches (i.e., points above the identity line, Extended Data Fig. 2h). This suggests that batch effects partly capture true biological effects. To confirm this, we estimated the proportion of variance explained by batches in EUB only (n = 212 out of 312 EIP samples, distributed on 4 out of 5 batches), which should estimate solely batch effects. Reassuringly, the correlation between the variance explained by batches in the uncorrected EUB data and that explained by continent of birth in the full batch-corrected data was low ($r_{\text{Continent}}$ = 0.043; Extended Data Fig. 2i). These results, together with the replication of PhIP-seq-based associations by Luminex immunoassays (Fig. 3d,h) and the observed differences in seroprevalence for viruses known to be more common in Africa than in Europe (Fig. 3b), strongly support the view that the observed effects of the continent of birth on antiviral antibody repertoires are not confounded by technical artifacts.

Evaluation of VirScan PhIP-seq data quality using ELISA- and Luminex-based assays

To validate the quality of the PhIP-seq data, we measured antibody reactivity against 55 antigens from 21 viruses using either ELISA (n = 12) or Luminex xMAP (n = 43) assays in all studied individuals from the MI cohort[3] (Methods). We compared PhIP-seq-based estimates of antibody reactivity to ELISA or Luminex serological results by first testing their association with 132 AVARDA breadth scores. We found that AVARDA breadth scores were specifically associated with the ELISA serostatus for the corresponding virus for 67% (8/12) of assays (Extended Data Fig. 3a), including herpesviruses such as CMV ($P_{\text{adj}} = 9.24 \times 10^{-160}$) and EBV ($P_{\text{adj}} = 2.72 \times 10^{-19}$) (Extended Data Fig. 3b,c), as well as the rubella ($P_{\text{adj}} = 4.43 \times 10^{-2}$) (Extended Data Fig. 3d) and varicella zoster ($P_{\text{adj}} = 3.73 \times 10^{-2}$) viruses. For the remaining assays, including IAV ($P_{\text{adj}} = 0.38$) and HBV ($P_{\text{adj}} = 0.63$), ELISA serostatus was associated with none of the AVARDA breadth scores (Extended Data Fig. 3e,f). Similarly, of the 43 Luminex assays, 62.8% (27/43) were significantly associated with an AVARDA breadth score, whereas 16 were not. Of the 27 significant associations, 77.8% (21/27) were

found with the cognate virus (Extended Data Fig. 3g). Akin to the ELISA assays, the viruses most significantly associated with Luminex-based serology included CMV ($P_{adj} = 1.50 \times 10^{-171}$) and EBV ($P_{adj} = 1.48 \times 10^{-42}$) (Extended Data Fig. 3h-i). Notably, the associations between the AVARDA scores and the Luminex-based serologies were stronger than those with ELISA-based serologies for several viruses, including measles, mumps, rubella, and influenza (Extended Data Fig. 3).

Several inherent methodological differences could explain the discrepancies observed between the PhIP-seq-based AVARDA breadth scores and the ELISA and Luminex assays: (i) the latter assays may target epitopes that are not the most immunoreactive, (ii) the AVARDA algorithm may discard the most immunoreactive peptides, (iii) the AVARDA algorithm may select cross-reactive peptides, and (iv) the VirScan assay may poorly measure reactivity against a specific epitope, due to the use of linear peptides or measurement errors. To evaluate these scenarios, we then tested the association between VirScan *Z*-scores for 2,608 public peptides and ELISA and Luminex serologies in the MI cohort (Methods). Associations between peptide *Z*-scores and serostatus were most significant for the cognate virus for 75% (9/12) of ELISA assays and 58% (25/43) of Luminex assays (Extended Data Fig. 4a-b). Serological reactivity to herpesviruses was again strongly associated between data sets. For example, 137 out of 180 VirScan peptides significantly associated with ELISA CMV serostatus originated from CMV ($P_{adj} = 7.84 \times 10^{-309}$). Similarly, 137 out of 169 VirScan peptides significantly associated with Luminex CMV serostatus originated from CMV ($P_{adj} = 2.02 \times 10^{-227}$) (Extended Data Fig. 4c-d). These results also revealed a few cases of cross-reactivity for the peptide-level PhIP-seq data. For example, CMV serostatus was significantly associated with several peptides from non-CMV viruses, including Enterovirus B ($P_{adj} = 8.35 \times 10^{-47}$), Tanapox ($P_{adj} = 3.83 \times 10^{-45}$), and Ebola ($P_{adj} = 4.34 \times 10^{-39}$) viruses.

Interestingly, there were cases where peptide *Z*-scores were strongly and specifically associated with ELISA- or Luminex-based serostatus, but the AVARDA score was not. For example, IAV peptide *Z*-scores were significantly associated with both ELISA- (71 significant peptides, min(P_{adj}) = 6.01×10^{-7}) and Luminex-based (137 significant peptides, min(P_{adj}) = 1.61×10^{-26}) IAV serostatuses (Extended Data Fig. 4e,f), whereas the AVARDA score for IAV was not (Extended Data Fig. 3e). This indicates that the peptide aggregation performed by the AVARDA algorithm can lead to false negatives and that increased resolution can be achieved by analyzing individual peptides.

VirScan also identified distinct reactivity patterns against different antigens from the same virus. For example, we found strong associations between ELISA-based EBV

serostatus for EA, VCA, and EBNA antigens and the corresponding VirScan peptides (Extended Data Fig. 4g). VirScan confirmed the known discordance between EBV serological markers[4], including a small number of VCA-positive, EBNA-negative individuals, and an even smaller group of VCA-negative, EBNA-positive individuals. Discordances among Luminex-based serologies were also apparent when comparing antibody titers against the nucleocapsid protein and the spike protein in some coronaviruses (Extended Data Fig. 4h). Collectively, these analyses show that VirScan PhIP-seq is overall specific and provides high resolution and sensitivity.

Serostatus prediction by machine learning outperforms heuristic methods

Defining serostatus from serological data, including PhIP-seq data, is a long-standing challenge because antibody titers often follow a continuous distribution and may target different antigens within the same virus. Previous studies using VirScan have defined seropositivity based on an arbitrary threshold of three to five positive peptides (or 'hits') without assessing the performance of this heuristic approach[5]. We thus leveraged the MI data to assess the prediction performance of three alternative approaches: (i) the hit-based heuristic method ('Hit-H method'), which assigns seropositivity for a given virus when the number of hits is > 3 or 5 (as in ref.[5]); (ii) the hit-based optimized method ('Hit-O method'), where we searched for the number of positive hits for a given virus that maximizes prediction precision and recall; and (iii) the AVARDA-based optimized method ('AVARDA-O method'), where we searched for the threshold value of the AVARDA breadth score for a given virus maximizes prediction precision and recall. Given the strong and specific associations observed between VirScan peptide *Z*-scores and gold-standard ELISA serostatuses (Extended Data Fig. 4), we also trained a predictive model of serostatus using logistic regression with Elastic Net penalty ('EN method'). Prediction performance was estimated by out-of-sample 5-fold cross-validation, keeping 30% of the MI data as the test set. As our primary objective was to provide a proof-of-concept that machine learning outperforms other approaches, we focused on predicting serostatus for four common viruses for which ELISA data were available: CMV, EBV (EA and EBNA), HSV-1, and HSV-2.

We found that the EN method outperformed the alternative approaches in almost all cases. For CMV, the machine-learning model showed a precision and recall of 97.8% and 97.8%, respectively (Table S2). We estimated a similar performance for the Hit-O method (95.7% and 98.9%), which uses 14 hits, whereas the Hit-H method, using 5 hits, yielded 36.6% precision and 100% sensitivity. The AVARDA-O method showed lower performance

than the EN and Hit-O approaches. For HSV-1, EN model precision and recall were 97.3% and 96.8%, versus 87.5% and 100% for the Hit-H approach. The Hit-O method slightly outperformed EN in terms of both precision and recall (Table S2). Differences in performance were more evident for HSV-2, with 93.9% precision and 92.4% recall for EN, while the alternative methods all showed <70% precision. Finally, EBV seropositivity (EBNA and EA epitopes) was predicted by EN with higher accuracy than any other method. Overall, these results indicate that the heuristic approach used in previous studies has relatively poor performance, particularly when the number of seropositive and seronegative samples is unbalanced. In contrast, predicting gold-standard serostatus using a machine-learning method trained on the VirScan data yields highly accurate results.

Socio-economic status and health biomarkers are weakly associated with the antibody repertoire

To identify demographic factors affecting the antiviral antibody repertoire, we searched for associations between VirScan peptide *Z*-scores and a curated list of 108 variables assessing socio-economic status (SES), health-related habits, medical history, and health biomarkers collected in the MI cohort (Table S3), while controlling for age, sex, and genetic structure (Methods). In addition to the strong effects of smoking behavior described (Fig. 4b-e), we found that 65 variables were significantly associated with antibody reactivity against at least one viral peptide ($P_{adj} < 0.05$; Fig. 4a).

Among the strongest associations, we found increased antibodies against enteroviruses in individuals who live with children ($P_{adj} = 0.0014$; Fig. 4a), who recently experienced a depressive episode ($P_{adj} = 2.45 \times 10^{-5}$), and whose highest diploma was high school ($P_{adj} = 0.0062$). High educational attainment was also associated with lower antibodies against HSV-1 ($P_{adj} = 0.0018$), confirming previous seroprevalence surveys[6]. Regarding health biomarkers, we detected a relatively strong, negative association between total protein levels and anti-RSV antibodies ($P_{adj} = 2.61 \times 10^{-5}$; Fig. 4a). Previous work has suggested that RSV infection induces a reduction in proteins, particularly surfactant proteins, which may contribute to RSV pathogenesis in the lung[7]. Additionally, we found that bilirubin levels were associated with lower antibody levels against HHV6A/B ($P_{adj} = 5.34 \times 10^{-5}$) and rhinoviruses ($P_{adj} = 8.82 \times 10^{-4}$), which remained significant after adjusting for smoking status ($P_{adj} = 2.50 \times 10^{-3}$). Lastly, mean corpuscular hemoglobin concentration was associated with anti-HSV-1 antibodies ($P_{adj} = 1.36 \times 10^{-4}$). Together, these findings suggest that antibody reactivity

against common viruses is weakly related to socio-economic status and several health biomarkers in a healthy population.

Latent infection by CMV and EBV affects immune cell parameters

Antibodies are produced by B cells, which vary in frequency with age, sex or comorbidities. To evaluate how the antibody repertoire relates to B-cell renewal and subtype frequencies, we tested the association between the *Z*-scores for 2,608 viral peptides and (i) the cellular proportion of 79 immune cell-types, (ii) the cell-surface expression of 88 differentiation markers[8] and, (iii) circulating counts of Kappa-deleting recombination excision circles (KREC; Methods), which quantify B-cell recent emigrants from the bone marrow[9]. No associations were found between PhIP-seq-based *Z*-scores and B-cell subtype proportions or markers of B-cell differentiation, nor with KREC numbers ($P_{adj} > 0.05$).

However, anti-CMV antibodies were associated with 15 immune parameters, 13 of which were T-cell-related. The strongest association was observed with the proportion of $CD4^+$ effector memory T-cells re-expressing CD45RA (T_{EMRA} cells; $P = 4.2 \times 10^{-69}$) (Extended Data Fig. 5a), confirming previous reports[8]. The CMV peptides significantly associated with $CD4^+$ T_{EMRA} cells originated from 47 Uniprot entries from 26 viral proteins. The strongest associations included peptides of various envelope glycoproteins, such as Glycoprotein B, Glycoprotein M, and Membrane glycoprotein UL139, but also tegument proteins such as Cytoplasmic envelopment protein 3 and pp150 (Extended Data Fig. 5b).

In addition to the established association between anti-CMV antibodies and T_{EMRA} cells, we identified significant associations between the surface expression of HLA-DR in conventional type 1 dendritic (cDC1) cells and antibody reactivity against 12 EBV peptides (Extended Data Fig. 5c; $P > 8.88 \times 10^{-9}$). All but one of the significant EBV peptides originate from the EBNA-LP antigen, a known modulator of viral gene expression. Together, these findings suggest that inter-individual variation in B-cell phenotypes do not correlate with humoral responses against specific viruses, and indicate instead that CMV and EBV latent infections modulate the activation and expansion of the T-cell compartment.

Supplementary References

1. Yu, Y., Mai, Y., Zheng, Y. & Shi, L. Assessing and mitigating batch effects in large-scale omics studies. *Genome Biol* **25**, 254 (2024).
2. Johnson, W. E., Li, C. & Rabinovic, A. Adjusting batch effects in microarray expression data using empirical Bayes methods. *Biostatistics* **8**, 118–127 (2007).
3. Scepanovic, P. *et al.* Human genetic variants and age are the strongest predictors of humoral immune responses to common pathogens and vaccines. *Genome Medicine* **10**, 59 (2018).
4. Klutts, J. S., Ford, B. A., Perez, N. R. & Gronowski, A. M. Evidence-Based Approach for Interpretation of Epstein-Barr Virus Serological Patterns. *J Clin Microbiol* **47**, 3204 (2009).
5. Xu, G. J. *et al.* Comprehensive serological profiling of human populations using a synthetic human virome. *Science* **348**, (2015).
6. Malkin, J.-E. *et al.* Seroprevalence of HSV-1 and HSV-2 infection in the general French population. *Sex Transm Infect* **78**, 201–203 (2002).
7. Kerr, M. H. & Paton, J. Y. Surfactant Protein Levels in Severe Respiratory Syncytial Virus Infection. *Am J Respir Crit Care Med* **159**, 1115–1118 (1999).
8. Patin, E. *et al.* Natural variation in the parameters of innate immune cells is preferentially driven by genetic factors. *Nat Immunol* **19**, 302–314 (2018).
9. van Zelm, M. C., Szczepanski, T., van der Burg, M., & van Dongen, J. J. Replication history of B lymphocytes reveals homeostatic proliferation and extensive antigen-induced B cell expansion. *J Exp Med* **204**, 645-655 (2007).
