# Supplementary material for: Demographic and genetic factors shape the epitope specificity of the human antibody repertoire against viruses
Source: Nat Immunol. 2026 Feb 16;27(3):600–12. doi: 10.1038/s41590-026-02432-7 (PMC12956577; doi:10.1038/s41590-026-02432-7)
Supplement: Supplementary file 2 — Reporting Summary [file 41590_2026_2432_MOESM2_ESM.pdf]

nature portfolio

Corresponding author(s): Etienne Patin

Last updated by author(s): Jan 5, 2026

Reporting Summary

Nature Portfolio wishes to improve the reproducibility of the work that we publish. This form provides structure for consistency and transparency in reporting. For further information on Nature Portfolio policies, see our Editorial Policies and the Editorial Policy Checklist.

Statistics

For all statistical analyses, confirm that the following items are present in the figure legend, table legend, main text, or Methods section.

n/a	Confirmed	
☐	☒	The exact sample size (*n*) for each experimental group/condition, given as a discrete number and unit of measurement
☐	☒	A statement on whether measurements were taken from distinct samples or whether the same sample was measured repeatedly
☐	☒	The statistical test(s) used AND whether they are one- or two-sided<br>*Only common tests should be described solely by name; describe more complex techniques in the Methods section.*
☐	☒	A description of all covariates tested
☐	☒	A description of any assumptions or corrections, such as tests of normality and adjustment for multiple comparisons
☐	☒	A full description of the statistical parameters including central tendency (e.g. means) or other basic estimates (e.g. regression coefficient) AND variation (e.g. standard deviation) or associated estimates of uncertainty (e.g. confidence intervals)
☐	☒	For null hypothesis testing, the test statistic (e.g. *F*, *t*, *r*) with confidence intervals, effect sizes, degrees of freedom and *P* value noted<br>*Give P values as exact values whenever suitable.*
☒	☐	For Bayesian analysis, information on the choice of priors and Markov chain Monte Carlo settings
☒	☐	For hierarchical and complex designs, identification of the appropriate level for tests and full reporting of outcomes
☐	☒	Estimates of effect sizes (e.g. Cohen's *d*, Pearson's *r*), indicating how they were calculated

Our web collection on statistics for biologists contains articles on many of the points above.

Software and code

Policy information about availability of computer code

Data collection	R v.4.3.0 with packages stats, pcaMethods v.1.94.0 and sva v.3.54.0; RStudio v.2024.04.2 Build 764.pro1; Bowtie v.2; Samtools; BWA-MEM; sambamba; GATK v.4.1; custom code available at https://github.com/h-e-g/virscan_association
Data analysis	R v.4.3.0 with packages tidyverse v.2.0.0, AVARDA v.1.0, GWASExactHW v.1.01, GWASTools v.1.48.0, topr v.1.1.10, relaimpo v.2.2-7 and DECIPHER v.2.30.0; RStudio v.2024.04.2 Build 764.pro1; Python v.3.12.2 with packages numpy v.1.26.4, scipy v.1.12.0, pandas v.2.2.1 and scikit-learn v.1.4.1; plink v.2; Eagle v.2.4; Minimac4; ConSurf-DB; PyMOL v.3.0.1; custom code available at https://github.com/h-e-g/virscan_association

For manuscripts utilizing custom algorithms or software that are central to the research but not yet described in published literature, software must be made available to editors and reviewers. We strongly encourage code deposition in a community repository (e.g. GitHub). See the Nature Portfolio guidelines for submitting code & software for further information.

Data

Policy information about availability of data

All manuscripts must include a data availability statement. This statement should provide the following information, where applicable:

- Accession codes, unique identifiers, or web links for publicly available datasets
- A description of any restrictions on data availability
- For clinical datasets or third party data, please ensure that the statement adheres to our policy

The VirScan3 PhIP-seq raw and processed data generated in this study have been deposited in the Institut Pasteur data repository, OWEY, which can be accessed via the following links: https://doi.org/10.48802/owey.84rn-jg72 (Milieu Intérieur) and https://doi.org/10.48802/owey.uCQ5VsxD (EvoImmunoPop). All association statistics obtained in this study can also be explored and downloaded from the web browser http://mirepertoire.pasteur.cloud/. All other pseudonymized datasets can be accessed on OWEY by submitting a data access request at https://redcap.pasteur.fr/surveys/?s=ND8TP8MDD3 (Milieu Intérieur) or https://redcap.pasteur.fr/surveys/?s=F3AA7J4M4W8LRNJ4 (EvoImmunoPop). The request will be reviewed by the respective data access committees (DAC). The DAC informs the research participants of the data access request and grants data access if the request is consistent with the informed consent signed by the participants. In particular, research on Milieu Intérieur and EvoImmunoPop datasets is restricted to research on the genetic and environmental determinants of human variation in immune responses. Data access is typically granted two months after request submission.

Research involving human participants, their data, or biological material

Policy information about studies with human participants or human data. See also policy information about sex, gender (identity/presentation), and sexual orientation and race, ethnicity and racism.

Reporting on sex and gender	This study evaluates how sex affects the human antibody repertoire against viruses. We report results by sex when appropriate and assess extensively how women's and men's antibody levels differ. Gender was not studied and self-reported gender was not collected.
Reporting on race, ethnicity, or other socially relevant groupings	This study evaluates how the continent of birth affects the human antibody repertoire against viruses. Participants from the EvoImmunoPop study are Belgian residents who were born in either sub-Saharan Africa (AFB) or Europe (EUB). The grouping was determined based on the self-declared place of birth of participants. Associations between antibody reactivity and the continent of birth were interpreted as statistical evidence for geographical differences in exposure to viruses between the two groups, rather than differences caused by race or ethnicity. AFB have been more exposed to herpesviruses, relative to EUB, whereas EUB have been more exposed to influenza A virus and rhinoviruses, owing to diverse climatic, ecological and/or health factors. This study also indicates that AFB and EUB individuals infected by the same virus differ in the viral peptides their antibodies target, implying that global serological surveys based on a single antigen may provide biased results.
Population characteristics	900 individuals from the Milieu Intérieur cohort were included in the present study, including 453 females and 447 males, aged from 20 to 69 years (Fig. 1a). 312 individuals from the EvoImmunoPop study were included, including 100 individuals born in Central Africa (AFB), and 212 born in Europe (EUB), all aged from 20 to 50 years (Fig. 1b).
Recruitment	Regarding the Milieu Intérieur cohort, recruitment was conducted in Rennes (France) in 2012-2013, with the aim to recruit 1,000 healthy individuals, including 500 women and 500 men stratified by age in 5 decades of age ([20–29], [30–39], [40–49], [50–59] and [60–69] years, with 200 subjects per stratum). A pre-existing donor database composed of ~110,000 donors was used for pre-screening potential participants in accordance with the study criteria. Additional advertising and website recruitment campaigns were launched in order to complete age and sex strata not sufficiently represented in the donor database. Eligibility was assessed by telephone interview and confirmed during a preliminary information meeting about the objectives of the research. Interested participants that met pre-screening criteria returned for the enrollment visit. During this visit, eligibility criteria were assessed in two stages: first, based on demographical data and clinical examination; and second, by analysis of blood and urine samples that were sent for clinical laboratory testing (Thomas et al., Clin Immunol 2015). Donors were excluded if they have evidence of, or report a history of, neurological, psychiatric or any severe/chronic/recurrent pathological conditions. Other exclusion criteria included a history or evidence of alcohol abuse, recent use of illicit drugs, recent vaccine administration, and recent use of immune modulatory agents. To avoid the influence of hormonal fluctuations in women during the peri-menopausal phase, only pre- or post-menopausal women were included. To avoid the presence of genetic structure in our study population, which would impact upon the power to detect genotype-to-phenotype associations, only individuals whose parents and grandparents were born in continental France were included. Regarding the EvoImmuoPop cohort, recruitment was performed at the Center for Vaccinology (CEVAC) of Ghent University Hospital (Ghent, Belgium). Sampling of related individuals was avoided because relatedness can confound genetic analyses. Individuals with serological signs of past or ongoing infection with human immunodeficiency virus (HIV), hepatitis B virus (HBV) or hepatitis C virus (HCV) were also excluded.
Ethics oversight	The Milieu Intérieur study has been approved by the Comité de Protection des Personnes — Ouest 6 (Committee for the Protection of Persons) and by the French Agence Nationale de Sécurité du Médicament (ANSM) and is sponsored by the Institut Pasteur (Pasteur ID-RCB Number: 2012-A00238-35). The study protocol, including inclusion and exclusion criteria for the Milieu Intérieur study, was registered on ClinicalTrials.gov under the study ID NCT01699893. The EvoImmunoPop study was approved by the ethics committee of Ghent University (Belgium, n° B670201214647) and the relevant French authorities (CPP, CCITRS and CNIL). The EvoImmunoPop study was also monitored by the Ethics Board of Institut Pasteur (EVOIMMUNOPOP-281297).

Note that full information on the approval of the study protocol must also be provided in the manuscript.

Field-specific reporting

Please select the one below that is the best fit for your research. If you are not sure, read the appropriate sections before making your selection.

☒ Life sciences ☐ Behavioural & social sciences ☐ Ecological, evolutionary & environmental sciences

For a reference copy of the document with all sections, see nature.com/documents/nr-reporting-summary-flat.pdf

Life sciences study design

All studies must disclose on these points even when the disclosure is negative.

Sample size	We found by simulations that the sample size of the primary cohort (Milieu Intérieur; n = 900) provides 95% power to detect a medium non-genetic or genetic effect, corresponding to 0.65 standard deviation of the peptide Z-score.
Data exclusions	As the present study focuses on human humoral responses against viruses, we discarded peptides from bacteria, fungi, and allergens from the VirScan library, resulting in 99,460 viral peptides. We also excluded VirScan data for peptides showing largely different Z-score values between experimental replicates. Discordant values were set to missing and peptides with >50% missing values were discarded. Finally, peptides with duplicated Uniprot entries were removed, leaving 97,975 peptides in the Milieu Intérieur dataset and 97,923 in the EvoImmunoPop dataset for the remaining analyses.
Replication	Measurement reproducibility was assessed by performing two replicates for each individual. Measurements with low reproducibility across individuals were excluded. All the results discovered in the Milieu Intérieur cohort were tested for replication in the EvoImmunoPop cohort, whenever possible. Associations with age were not replicated for a few viruses and peptides, likely because of the smaller size of the replication cohort (EvoImmunoPop; n = 312). Associations with sex were not tested for replication, as the replication cohort includes males only. Associations with the continent of birth were tested in the EvoImmunoPop cohort only, as the Milieu Intérieur cohort includes only participants born in France. Genetic associations were all tested for replication, except at the IGH and IGK loci, which were sequenced in the Milieu Intérieur cohort only.
Randomization	Prior to PhIP-seq experiments, samples were distributed in processing plates according to sample identifiers. As sample identifiers were partially correlated with age and continent of birth, and the PhIP datasets were affected by plate effects (Supplementary Fig. 1), VirScan Z-scores were corrected for plate effects using the ComBat function from the sva R package, providing age and continent-of-birth variables as biological factors with true effects on the data.
Blinding	Investigators were blinded to group allocation during data collection.

Reporting for specific materials, systems and methods

We require information from authors about some types of materials, experimental systems and methods used in many studies. Here, indicate whether each material, system or method listed is relevant to your study. If you are not sure if a list item applies to your research, read the appropriate section before selecting a response.

Materials & experimental systems

n/a	Involved in the study	
☒	☐	Antibodies
☒	☐	Eukaryotic cell lines
☒	☐	Palaeontology and archaeology
☒	☐	Animals and other organisms
☐	☒	Clinical data
☒	☐	Dual use research of concern
☒	☐	Plants

Methods

n/a	Involved in the study	
☒	☐	ChIP-seq
☒	☐	Flow cytometry
☒	☐	MRI-based neuroimaging

Clinical data

Policy information about clinical studies

All manuscripts should comply with the ICMJE guidelines for publication of clinical research and a completed CONSORT checklist must be included with all submissions.

Clinical trial registration	NCT01699893
Study protocol	The Milieu Intérieur study protocol is described in Thomas et al., Clin Immunol 2015. The EvoImmunoPop study protocol is described in Quach*, Rotival*, Pothlichet*, Loh* et al., Cell 2016.
Data collection	Blood samples were collected from the Milieu Intérieur healthy, fasting donors every working day from 8AM to 11AM, from September 2012 to August 2013, at Biotrial (Rennes, France). Tracking procedures were established in order to ensure delivery to Institut Pasteur (Paris) within 6 hours of blood draw. Upon receipt, samples were kept at room temperature until plasma preparation

and DNA extraction. Blood samples were collected from the EvoImmunoPop donors on EDTA-blood collection tubes at the Center for Vaccinology (CEVAC) of Ghent University Hospital (Ghent, Belgium), from 2012 to 2013.

Outcomes

The aim of the present study was to evaluate how demographic and genetic factors affect the human antibody repertoire against viruses. The primary outcomes of this study were the quantitative measures of antibody reactivity against peptides of all known viruses infecting humans. To assess these measures, we employed PhIP-Seq using the VirScan V3 library, a viral peptidome scanning method based on bacteriophage display and immuno-precipitation. The phage library was incubated with plasma samples normalized to total IgG concentration and controls (bead samples) to form IgG-phage immunocomplexes. The immunocomplexes were then captured by magnetic beads, lysed, and sent to next-generation sequencing. The secondary outcomes were demographic variables, including age, sex, health-related habits, and vaccination and medical history, which were obtained through structured questionnaires, and genetic variables, which were obtained by SNP array genotyping and genotype imputation.

Plants

Seed stocks

n/a

Novel plant genotypes

n/a

Authentication

n/a
